# A high prevalence of *Cutibacterium acnes* infections in scoliosis revision surgery, a diagnostic and therapeutic dilemma

**DOI:** 10.1007/s43390-022-00599-1

**Published:** 2022-10-25

**Authors:** Stefan J. Gelderman, Christhoper Faber, Greetje A. Kampinga, Paul C. Jutte, Joris J. W. Ploegmakers, Andor W. J. M. Glaudemans, Marjan Wouthuyzen-Bakker

**Affiliations:** 1 Department of Orthopaedic Surgery, University of Groningen, University Medical Center Groningen, Groningen, The Netherlands; 2 Department of Medical Microbiology and Infection Prevention, University of Groningen, University Medical Center Groningen, Groningen, The Netherlands; 3 Medical Imaging Center, Department of Nuclear Medicine and Molecular Imaging, University of Groningen, University Medical Center Groningen, Groningen, The Netherlands

**Keywords:** Spinal instrumentation infection, Revision surgery, Diagnostics, Treatment

## Abstract

**Purpose:**

To investigate if serum inflammatory markers or nuclear imaging can accurately diagnose a chronic spinal instrumentation infection (SII) prior to surgery.

**Methods:**

All patients who underwent revision of spinal instrumentation after a scoliosis correction between 2017 and 2019, were retrospectively evaluated. The diagnostic accuracy of serum C-reactive protein (CRP) and Erythrocyte Sedimentation Rate (ESR), ^18^F-fluorodeoxyglucose positron emission tomography combined with computed tomography (FDG–PET/CT) and Technetium-99m-methylene diphosphonate (99mTc-MDP) 3-phase bone scintigraphy (TPBS) to diagnose infection were studied. Patients with an acute infection or inadequate culture sampling were excluded. SII was diagnosed if ≥ 2 of the same microorganism(s) were isolated from intra-operative tissue cultures.

**Results:**

30 patients were included. The indication for revision surgery was pseudoarthrosis in the majority of patients (*n = *15). 22 patients (73%) were diagnosed with SII. In all infected cases, *Cutibacterium acnes* was isolated, including 5 cases with a polymicrobial infection. The majority of patients had low inflammatory parameters preoperatively. For CRP > 10.0 mg/L, the sensitivity was 9.1% and specificity 100%; for ESR > 30 mm/h, the sensitivity was 9.1% and specificity 100%. The diagnostic accuracy for nuclear imaging was 64% for the FDG–PET/CT and 67% for the TPBS to diagnose infection.

**Conclusions:**

The prevalence of SII in patients undergoing revision spinal surgery is high, with *Cutibacterium acnes* as the main pathogen. No diagnostic tests could be identified that could accurately diagnose or exclude SII prior to surgery. Future studies should aim to find more sensitive diagnostic modalities to detect low-grade inflammation.

## Introduction

Scoliosis is a three-dimensional deformity of the spine that can lead to cosmetic concerns, chronic back pain and even respiratory problems [1, 2]. Surgical correction is indicated in patients with a Cobb angle greater than 50 degrees or when the curvature is likely to progress [3]. The goal of surgical treatment is to achieve solid fusion with correction of spine deformity. A spinal instrumentation infection (SII) is considered a major complication of surgery and has been reported in approximately 3% of spinal deformity corrections [4]. Low-grade infections may lead to implant loosening and/or chronic pain, and mandate revision surgery [5, 6]. As the presence of an infection requires a different surgical strategy (i.e., thorough debridement) and immediate start of antibiotic peroperatively, it is important to diagnose a SII prior to revision surgery. However, it is unknown which specific tests can diagnose or exclude an infection in spinal instrumentation [7]. Unlike in prosthetic joints, where synovial fluid can be aspirated to accurately exclude an infection in joint implants using cytology, biomarkers and/or culture [8, 9], there is no fluid to aspirate in spines. For this reason, one can only rely on serum markers and/or imaging as a non-invasive diagnostic approach to diagnose infection. At the moment, no diagnostic flowchart exists for diagnosing a SII prior to surgery, and evidence for the use of imaging for this indication is scarce. The aim of our study was to determine the diagnostic accuracy of serum inflammation markers and two types of nuclear imaging (i.e., ^18^F-fluorodeoxyglucose positron emission tomography combined with computed tomography (FDG–PET/CT) and Technetium-99 m-methylene diphosphonate (99mTc-MDP) 3-phase bone scintigraphy (TPBS)) to diagnose a chronic SII in scoliosis patients prior to revision surgery.

## Patients and methods

### Patients

All patients who underwent spinal revision surgery between January 2017 and December 2019 were retrospectively evaluated. Patients were excluded when they were within 3 months after the primary surgery and/or if less than 4 cultures (including sonication) were obtained during revision surgery [10]. In addition, patients who underwent revision surgery because of an acute haematogenous infection were also excluded. A haematogenous infection was defined as an acute onset of symptoms in a prior asymptomatic back, combined with evidence of bacteraemia and increased C-reactive protein [11]. All surgeries were performed at one university hospital by two experienced orthopaedic spine surgeons. An average of 60 primary scoliosis correction surgeries are performed in our centre each year.

### Data collection

Patients’ data were retrospectively collected, and included clinical symptoms, preoperative serum inflammatory markers (C-reactive protein [CRP] and Erythrocyte Sedimentation Rate [ESR]), results of preoperative X-rays, CT scans, FDG–PET/CT scan and ^99m^Tc-MDP TPBS, surgical findings, intraoperative cultures and histology. Before 2018, nuclear imaging was routinely performed using ^99m^Tc-MDP TPBS. From 2018, ^99m^Tc-MDP TPBS has been substituted by the FDG–PET/CT for this indication. Nuclear imaging was only performed in cases with a clinical suspicion of a chronic infection and/or to differentiate between septic and aseptic loosening. Pre-operative X-rays and CT scans were taken into account in the pre-operative diagnosis of instrumentation failure and/or loosening. Serum inflammatory markers closest to the revision surgery were used in the analysis.

### Definition of spinal instrumentation infection and microbiology

Tissues samples were strategically obtained at different levels of the spinal instrumentation at the tissue instrumentation interface. During the procedure, cultures were also obtained from the most macroscopic suspected tissues. For each sample, clean instrumentation was used. No cultures were taken from subcutaneous tissues, fistulas or superficial wounds. Each sample was cultured for 9–14 days, depending on the clinical suspicion of a low-grade infection [12]. Samples were cultured in fastidious broth, on blood and chocolate agar under aerobic conditions and on Brucella blood agar under anaerobic conditions (Mediaproducts, Groningen, Netherlands). In addition, extracted spinal instrumentation was sonicated for 1 min at 40 kHz in an ultrasonic bath with sterile Ringer’s lactate to yield bacteria embedded in biofilm. The sonication fluid was subsequently incubated in blood culture bottles and 100 μL was cultured on blood, chocolate, and Brucella blood agar. A SII was diagnosed when the same micro-organism with identical antibiogram was isolated in two or more intra-operative cultures.

### Imaging acquisition

All FDG–PET/CT scans were performed with an EARL accredited scanner, Siemens Biograph MCT 64 or 40 slice (Knoxville, TN, USA). Patients were instructed to fast for at least 6 h prior to the administration of FDG. Blood glucose levels were measured before injection of the FDG and had to be below 11 mmol/L. The acquisition of the scan started approximately 60 min after 3 MBq/kg FDG administration. All scan acquisition parameters were according to guidelines from the European Association of Nuclear Medicine (EANM) [13] and the images were evaluated by a nuclear medicine physician using pre-defined interpretation criteria. The scan was considered positive with the presence of either heterogeneous and/or focal increased FDG uptake near the spinal instrumentation.

Patients who underwent a TPBS received 700 MBq ^99m^Tc-MDP intravenously. Phase 1 (perfusion phase) started immediately after the injection and consisted of 2 min. The second phase (diffusion phase) started at the third minute until the fifth minute. The third phase started 3 h after the injection in which a static image was taken in anterior posterior position. The TPBS was considered positive for infection when increased focal uptake was present in the third phase near the spinal instrumentation in comparison to other bones (background).

### Statistics

IBM SPSS statistics version 26 (IBM Corp, Armonk, NY, USA) was used for statistical analysis. Descriptive statistics were calculated. Continuous variables are presented as median and range. Differences between groups were calculated with the Mann–Whitney *U* test. The diagnostic accuracy, sensitivity, specificity, positive predictive value (PPV), negative predictive value (NPV) were calculated with the per-operative cultures as reference for infection as indicated in the definition for SII in the text above. Inconclusive results were not included in the diagnostic analysis.

### Ethics, funding, and potential conflicts of interest

A waiver was obtained for this study from the UMCG medical ethical committee on 3 December 2019 (number 201900774). None of the authors had funding that might pose a conflict of interest in connection with the submitted article.

## Results

### Patient characteristics

A total of 37 scoliosis patients underwent an extraction with or without reimplantation of spinal instrumentation between 2017 and 2019. 7 patients were excluded from the study: 3 patients were diagnosed with an infection within 3 months after surgery and in 4 patients inadequate culture sampling was performed, leaving a total of 30 patients for the final analysis. Table 1 shows an overview of the included patients. The indication for extraction of spinal instrumentation was pseudoarthrosis (*n = *15) in the majority of patients, combined with loosening of the instrumentation in 7 patients and instrumentation failure (e.g., broken screws or rods) in 7 patients. 3 patients were pre-operatively suspected for a chronic SII. The primary surgery of the included patients was performed between 2008 and 2017. All patients underwent a posterior surgical approach, using titanium and cobalt chrome instrumentation. The Quantum Spinal Rod system (Pioneer surgical technology) was used until 2012 (7 patients) and since the year 2012 the Mesa 2 Deformity (Stryker) instrumentation (23 patients) is in use. The group consisted of 26 females and 4 males with a mean age at revision surgery of 19.9 years (SD ± 3.5). The mean time between the primary and revision surgery was 4.4 years (SD ± 2.4). 22 out of 30 patients were diagnosed with a SII (73%). 20 out of the 22 infected patients had at least 3 positive intraoperative cultures with *C. acnes*. Five SIIs were polymicrobial (16%), with *Staphylococcus epidermidis* as the most common additional microorganism isolated.

**Table 1:** Patient data

Patient	Sex	Indication for revision surgery	CRP (mg/L)	ESR (mm^3^/h)	WBC × 10^9^/L	Bone-scan	FDG–PET /CT scan	Histology	Per-operative loosening	Cultures (positive)	Sonication culture	Spinal Instrumentation Infection
1	V	Pseudoarthrosis and loosening	8.0	4	11.6	–	Positive	Negative	Negative	*C. acnes* (2 of 8)	Negative	Yes
2	V	Suspected infection	28	57	7.8	–	Positive	Positive	Positive	*C. acnes* (9 of 9) *S. epidermidis* (1 of 9)	*S. epidermidis*	Yes
3	V	Hardware failure	0.4	3	8.5	–	–	Negative	Positive	*C. acnes* (3 of 5) *S. epidermidis* (1 of 5) *S. saccharolyticus* (2 of 5)	*S. epidermidis*	Yes
4	V	Loosening	0.3	1	6.7	–	Positive	Negative	Positive	*C. acnes* (3 of 6)	*C. acnes*	Yes
5	V	Suspected infection	1.5	3	8.2	–	–	Negative	Positive	*C. acnes* (4 of 4)	*S. capitis* *Dermacoccus species* *S. saccharolyticus* *C. acnes*	Yes
6	M	Loosening	1.5	4	6.9	Negative	–	Negative	Positive	*C. acnes* (6 of 6)	*C. acnes* *C. granulosum*	Yes
7	V	Suspected infection	3.2	16	10.4	Negative	–	Inconclusive	-	*C. acnes* (4 of 6) *S. epidermidis* (1 of 6)	*S. epidermidis*	Yes
8	V	Hardware failure	20	33	3.3	Positive	Positive	Negative	Positive	*C. acnes* (6 of 7) *S. epidermidis* (3 of 7) *C. granulosum* (2 of 7)	*C. acnes* *S. epidermidis*	Yes
9	V	Hardware failure	7.9	5	7.2	–	–	Negative	Negative	*C. acnes* (4 of 4)	*C. acnes*	Yes
10	V	Infection	1.1	9	6.1	Positive	–	–	–	*C. acnes* (6 of 6)	*C. acnes* *S. capitis*	Yes
11	V	Pseudoarthrosis and hardware failure	2.6	5	6.3	Negative	–	Negative	Negative	*C. acnes* (3 of 3)	*C. acnes*	Yes
12	V	Pseudoarthrosis hardware failure	4.2	2	6.8	Positive	–	Negative	Positive	*C. acnes* (5 of 5)	*C. acnes* *S. epidermidis*	Yes
13	V	Hardware failure	2.6	7	8.7	Negative	–	–	–	*C. acnes* (4 of 5)	*C. acnes*	Yes
14	M	Hardware failure	3.5	3	8.6	–	–	Negative	–	*C. acnes* (7 of 8) *S. epidermidis* (4 of 8)	*C. acnes* *S. epidermidis*	Yes
15	V	Pseudoarthrosis and loosening	0.3	3	7.4	–	–	Negative	Negative	*C. acnes* (2 of 6)	*S. capitis*	Yes
16	V	Pseudoarthrosis and loosening	1	3	5.0	Positive	Negative	Negative	Positive	*C. acnes* (2 of 5)	*C. acnes*	Yes
17	M	Pseudoarthrosis and hardware failure	0.8	2	5.4	–	Positive	Negative	Positive	*C. acnes* (4 of 5)	Negative	Yes
18	V	Pseudoarthrosis and loosening	8	7	8.7	–	Negative	Negative	Positive	*C. acnes* (4 of 5)	*C. acnes* *Dermacoccus nishinomiyaensis* *Micrococcus luteus*	Yes
19	V	Pseudoartrosis and loosening	0.3	8	8.0	–		Negative	Positive	*C. acnes* (3 of 4) *S. epidermidis* (1 of 4)	*C. acnes*	Yes
20	V	Suspected infection	0.4	10	5.5	–	Negative	Negative	Negative	*C. acnes* (1 of 6)	*C. acnes*	Yes
21	M	Pseudoarthrosis and hardware failure	3.4	18	10.6	–	–	–	Negative	*C. acnes* (2 of 4)	*C. acnes*	Yes
22	V	Pseudoarthrosis and loosening	0.4	7	7.1	–	Negative	Negative	Positive	*C. acnes* (4 of 7)	*C. acnes*	Yes
23	V	Pseudoarthrosis and hardware failure	0.4	2	5.8	–	Negative	Negative	Negative	Negative	*C. acnes* *C. granulosum*	No
24	V	Suspected infection	1.5	17	10	–	Negative	Negative	Negative	Negative	Negative	No
25	V	Hardware failure	3.7	5	9	–	Positive	Negative	Positive	*C. afermentans* (1 of 5) *S. capitis* (1 of 5)	*C. acnes*	No
26	V	Pseudoartrosis and loosening	3.1	9	10.9	–	Negative	Negative	Positive	Negative	Negative	No
27	V	Pseudoartrosis and hardware failure	6	8	5.9	–	–	Negative	Positive	Negative	Negative	No
28	V	Pseudoartrosis and hardware failure	1	4	8.6	–	–	Negative	Positive	*C. acnes* (1 of 8)	Negative	No
29	V	Hardware failure	6	-	10.0	Negative	–	Negative	Negative	*Micrococcus luteus* (1 of 5)	Negative	No
30	V	Pseudoartrosis and loosening	1.2	7	7.9	–	Negative	Negative	Positive	Negative	Negative	No

### Diagnostic tests

Table 2 shows the diagnostic accuracy of different preoperative and intra-operative diagnostic tests.

**Table 2:** Diagnostic accuracy of different pre-operative and intra-operative diagnostic tests

Pre-operative tests						Intra-operative tests	
	CRP > 5.0 mg/L (*n = *30) (%)	CRP > 10 mg/L (*n = *30) (%)	ESR > 30 mm/h (*n = *29) (%)	FDG–PET scan (*n = *14) (%)	Bone-scintigraphy (*n = *9) (%)	Histology (*n = *26) (%)	Per-operative Loosening (*n = *26) (%)
Sensitivity	22.7	9.1	9.1	55.5	50.0	5.6	66.7
Specificity	62.5	100	100	80.0	100	100	37.5
PPV	62.5	100	100	83.3	100	100	70.6
NPV	22.7	28.6	25.9	50.0	20.0	32	33.3
Accuracy	33.3	33.3	31.0	64.3	66.6	34.6	57.7

#### Pre-operative tests

The median serum CRP level of patients diagnosed with a SII was 2.1 mg/L (range: 0.3–28) versus 2.3 mg/L (range 0.4–6.0) for those without an infection (*P = *0.778). Using the generally accepted cutoff value of 10 mg/L to diagnose infection, [14] a diagnostic accuracy of 33% was calculated, with a corresponding sensitivity of 9% and specificity of 100%. Only 2 out of 22 patients diagnosed with a SII had a CRP > 10 mg/L (9%), resulting in a low negative predictive value to exclude infection. Lowering the CRP cutoff value to 5 mg/L did not improve its diagnostic accuracy. Similar results were observed for ESR, showing a 100% specificity but very low sensitivity for infection diagnosis (Table 2).

Out of the total cohort, 14 patients underwent a preoperative FDG–PET/CT scan and 9 patients a TPBS to diagnose or exclude a SII, resulting in an overall diagnostic accuracy of 64% and 66%, respectively. Both imaging modalities had a high specificity, in which the bone scintigraphy appeared most specific, but both modalities had a low negative predictive value to exclude infection (50% for FDG–PET/CT versus 20% for bone-scintigraphy).

#### Intra-operative tests

We additionally analyzed perioperative markers to diagnose SII (Tables 1 and 2). In 27 out of 30 cases, tissue samples for histology were obtained. In only 1 out of the 18 patients with a SII (5.6%), neutrophil influx was observed by the pathologist. This particular patient had a poly-microbial infection with *C. acnes* and *S. epidermidis*. All other SII cases had false negative histology (except 1 inconclusive result), resulting in an overall diagnostic accuracy of histology samples of 35%.

Per-operative loosening of the spinal instrumentation observed by the orthopaedic surgeon during surgery had an overall diagnostic accuracy for SII of 58%, with a moderate positive predictive value of 71% but low negative predictive value of 33%.

## Discussion

We investigated the diagnostic accuracy of serum inflammatory markers and nuclear imaging to diagnose a chronic SII prior to surgical extraction of the instrumentation. All diagnostic methods we evaluated showed poor diagnostic performance to rule out an infection. More than 75% of patients diagnosed with a SII had a serum CRP level of < 5 mg/L, and nuclear imaging was false negative in 50% of cases. Although a serum CRP > 10 mg/L and an ESR > 30 mm/h had a very high PPV to diagnose SII, only a small minority of patients with a SII had inflammatory parameters above this threshold.

The high specificity, but poor sensitivity of serum inflammatory markers that we observed in our study is probably due to the low virulence of the most frequent isolated microorganism found in our study: *C. acnes*. This finding has been confirmed by others: Akgün and colleagues [15] found that the most optimal cutoff level for serum CRP to diagnose a chronic SII after instrumented spinal fusion was 4 mg/L, but this threshold still resulted in a poor sensitivity and specificity of 64% and 68%, respectively. The same finding has been reported in chronic periprosthetic joint infections (PJI) of the shoulder in which, just as in spinal infections, *C. acnes* plays the most prominent role as causative pathogen. Pottinger et al. [16] found that only 17% of patients with a chronic PJI of the shoulder had a CRP > 10 mg/L, and ESR was elevated in merely 20% of the infected cases using a cutoff of > 15 mm/h. Two other studies reported similar findings in shoulder PJIs caused by *C. acnes* [17, 18], reporting very poor sensitivities for serum CRP, ESR and interleukin-6.

Even though FDG–PET/CT and bone scintigraphy have shown a very high sensitivity in a recent meta-analysis to diagnose fracture related infections [19], in our analysis, both types of imaging failed to detect SII when caused by *C. acnes*. Probably due to the low-grade inflammation caused by *C. acnes*, these nuclear imaging modalities lack diagnostic power, as demonstrated also for chronic shoulder PJIs [20]. In the near future, nuclear imaging techniques will be available with the potential to diagnose low grade infections more accurately (e.g., total body PET and PET combined with CT iterative metal artefact reduction (iMAR) [21, 22].

The high rate of positive cultures we found in our study is in contrast with findings in literature. To date, studies in primary shoulders without infection demonstrate that around 20% of patients harbour *C. acnes* in the deep layers of the skin [23–26]. In most of these studies, two or more cultures were positive. In addition, two studies described the rate of colonization in spinal instrumentation. Shifflet and colleagues [27] found positive intraoperative cultures in presumed aseptic revisions of the spine in 20% of cases, and Shiban et al. [6] in 42% of the cases with loosened screws against 18% in non-loosened screws. The high incidence of *C. acnes* infection in our cohort may be due to the young population studied, having a higher rate of *C. acnes* colonization compared to older adults [28–30].

Due to the current lack of highly sensitive techniques to diagnose SII prior to surgery, it is reasonable to start empirical antibiotic treatment per-operatively when the spinal instrumentation is revised to prevent new bacterial colonization and biofilm formation of the new spinal implant. The empirical treatment should at least cover *C. acnes*, which was the most causative pathogen in our analysis in scoliosis patients. Alternatively, preoperative tissue biopsies could be obtained to diagnose infection prior to re-instrumentation surgery. However, this is an invasive procedure, and it is hard to determine the hot spots to biopsy. Another strategy is to identify patients who are at highest risk for a spinal infection to decide whether empirical antibiotic treatment should be started or if this can be withheld. For example, it is known that testosterone increases the skin surface lipids and consequently increases *C. acnes* colonization of the skin [31]. Indeed, studies show that especially young, healthy males with a low BMI and those taking testosterone supplements are at highest risk to develop an infection with *C. acnes* [28, 32]. Unfortunately, we were not able to identify these risk factors in our small cohort and most included patients were female.

Even more important, it is clear from our analysis, demonstrating a very high rate of SII due to *C. acnes* in scoliosis patients, that antibiotic prophylaxis in this patient category should be optimized. Current perioperative cefazolin prophylaxis and topical disinfectants fail in eliminating *C. acnes* from the surgical site [33], as the pathogen resides deep in the skin and, therefore, survives topical disinfectants [34]. Currently, there are no clear guidelines in eradicating *C. acnes* from the surgical site [35]. In dermatology there is experience in eradicating *C. acnes* in patients with acne vulgaris [36], by the use of benzyl peroxide and topical antibiotics (clindamycin 1% and erythromycin 2%) [37]. Future studies are necessary to demonstrate whether this would be beneficial as a pre-operative prophylaxis measurement.

There are several limitations to our study. First, in 2017 more revision surgeries were performed due to an increase awareness of low-grade infections as a cause of chronic pain, which may be the reason for the high infection rate in the studied period. Second, the number of included patients is small and not all patients underwent nuclear imaging. Nevertheless, as all analysed diagnostic tests showed false negative results in a significant number of cases, we do not expect that these results will be different with a higher sample size. Second, it can be questioned whether failure of the hardware was indeed caused by an infection in those cases that were classified as infected, or if mechanical failure also have played a crucial role. This brings us to the ongoing debate whether the presence of *C. acnes* in the deeper tissues are indeed pathogens or mere colonizers. Unfortunately, histology could not provide the distinction between infection or colonization either. In our analysis, intraoperative histology samples did not show signs of infection in most cases as judged by the pathologist, which is consistent with findings in literature [17, 38]. New available techniques such as amplicon based sequencing, shotgun metagenomics and proteonomics may help us to better differentiate between infection and colonization. However, it is important to note that in our study, that the majority of patients had 4 or more positive cultures with *C. acnes*, and since we have strict protocols on tissue sampling and processing, we consider colonization instead of infection less likely. In addition, all patients had clinical signs of infection, e.g., chronic pain, pseudoarthrosis, implant loosening and/or failure.

In conclusion, *C. acnes* plays a prominent role in chronic SII and non-invasive methods to sensitively diagnose the presence of a chronic SII prior to revision surgery are lacking. Future studies should aim to find more sensitive diagnostic modalities to detect low-grade inflammation in spinal implants, improve antibiotic prophylaxis and should decipher the difference between *C. acnes* colonization and infection.

## Data Availability

The data sets generated during and analysed during the current study are available from the corresponding author on reasonable request.
